# A planned hybrid culotte stenting procedure in the setting of an acute STEMI

**DOI:** 10.1186/1757-1626-2-9104

**Published:** 2009-11-27

**Authors:** Chitradeep De, Medhat Zaher, Mayur Lakhani, Joseph T McGinn, Roberto Baglini, Duccio Baldari

**Affiliations:** 1 Staten Island University Hospital, 475 Seaview Avenue, Staten Island, New York 10305, USA; 2 University of Pittsburgh Medical Center in Italy, Via Tricomi 1, 90127 Palermo, Italy

## Abstract

**Introduction:**

Bifurcation lesions have traditionally presented a unique problem for interventional cardiologists because of their inherent anatomy and risk of closure of the side branch, after a percutaneous intervention for the primary lesion of the main branch.

**Case Presentation:**

We report the case of a 57-year-old man who presented with acute ST-segment elevation myocardial infarction secondary to a 100% occlusion at the ostium of first diagonal (D1) branch. Patient also had a 70% stenosis of the mid-segment of the left anterior descending (LAD) coronary artery at the D1 branching point (1,1,1 Medina classification). A bare metal stent (BMS) was deployed at the site of the culprit lesion in the D1, while a drug eluting stent (DES) was placed in the LAD. We believe that the BMS at the culprit thrombotic, inflamed site in D1 is more likely to re endothelialize than a DES and the DES in the LAD, is less likely to re-stenose than a BMS.

**Conclusion:**

This is the only reported case, where in the setting of an acute ST elevation myocardial infarction, a hybrid Culotte technique was successfully performed with excellent long-term results, thus achieving an acceptable balance of risks between restenosis (in the case of a BMS) and stent thrombosis (in the case of a DES).

## Case

A 57-year-old Caucasian man presented to the emergency room with severe precordial chest pressure radiating to the left jaw and arm and associated with dyspnea and nausea. The past medical history included gout and hypercholesterolemia for which he was treated with atorvastatin and allopurinol. He also was a chronic heavy smoker but denied history of alcohol or drug abuse.

The patient was in mild distress. Initial physical examination revealed blood pressure of 180/115 mmHg and heart rate of 96 beats/min with regular rhythm. Cardiac examination showed normal first and second heart sounds with no audible murmurs, rubs or gallops. He was given sublingual nitroglycerine with some relief of chest pain. A stat electrocardiogram revealed ST-segment elevation in leads V3-V6. He was given aspirin 325 mg orally, metoprolol 50 mg orally, clopidogrel 300 mg orally, atorvastatin 80 mg orally, a bolus of heparin 5000 units intravenously as well as nitroglycerine infusion at 25 mcg/min.

Emergent selective coronary angiography showed a 100% occlusion with a filling defect suggestive of thrombosis at the ostium of the first diagonal branch (D1) and a 70% stenosis of the mid-segment of the left anterior descending coronary artery (LAD) at the D1 branching point (1,1,1 Medina classification). Balloon angioplasty was performed on the D1 lesion using Maverick 2 RX 2.0 × 12 balloon with maximum inflation pressure of 16 atm leading to reestablishment of blood flow through D1. Chest pain improved significantly and ST-segment elevations started to regress. Further dilation of the same lesion was done using Maverick 2 RX 2.5 × 15 balloon with maximum inflation pressure of 6 atm. A Mini Vision 2.5 × 18 bare metal stent (BMS) was deployed in the D1 ostium with maximum inflation pressure of 16 atm.

Using the Cullote technique, balloon angioplasty using Maverick 2 RX 2.5 × 15 balloon with maximum inflation pressure of 10 atm was performed on the 70% stenosis of the mid LAD. Then a Paclitaxel eluding stent (Taxus) RX 3.0 × 20 was deployed in the LAD lesion with maximum inflation pressure of 16 atm. Simultaneous kissing balloons were inflated in the D1 and the mid LAD using Maverick 2 RX 2.5 × 15 with maximum inflation pressure of 6 atms in the LAD and Maverick 2 RX 2.5 × 15 with maximum inflation pressure of 10 atms in D1. Following intervention, there was excellent angiographic appearance with 0% residual stenosis of both vessels. The patient was transferred to the coronary care unit for overnight observation and was discharged home the following day without complications.

Three months later, staged percutaneous coronary intervention of an 80% stenosis of the mid right coronary artery was performed. Angiography revealed a 40% restenosis of the BMS in the D1 and no restenosis in drug eluting stent (DES) in the LAD. The patient continues to be asymptomatic.

## Discussion

Interventional treatment of coronary artery bifurcation lesions represents one of the most challenging techniques in the field of coronary interventions. Bifurcation lesions represent up to 16% of coronary targets for intervention [1]. When compared with non-bifurcation interventions, bifurcation interventions have a lower rate of procedural success, higher procedural costs, longer hospitalization, and a higher rate of clinical and angiographic re-stenosis. Plaque redistribution "plaque shift" across the carina of the bifurcation may risk occlusion of the side branch or even the parent vessel [2-5]. Eccentric stenosis at the bifurcation and ostial narrowing of the side branch further increase this risk [4]. The kissing balloon technique was developed more than twenty years ago for side branch protection [6]. More recently, in the coronary artery stents era, many techniques were described to stent bifurcation lesions [7]. However, it was shown that stenting both vessels in a bifurcation lesion provides no advantage in terms of procedural success and late outcome versus a simpler strategy of stenting only the parent vessel if feasible anatomically [8]. In some cases, the bifurcation lesion anatomy places a major vessel or more at a very high risk of occlusion dictating stenting both vessels. Chevalier et al have first proposed the Culotte technique as a new option for bifurcation lesion stenting in 50 patients and reported an acceptable re-stenosis rate of 24% [9].

However, later reports on double stenting of bifurcation lesions using BMS although provides better immediate angiographic results is associated with higher (37.6%) incidence of clinically driven repeat target lesion revascularization with 2 stents as compared to 5.6% using 1 stent at six months [1]. On the other hand, bifurcation lesion stenting using DES was shown to be associated with significantly higher rates of stent thrombosis in multiple studies [10-13]. In one study, DES was associated with a significantly higher risk of stent thrombosis at 9 months with a hazard ratio of 6.42 (95% CI, 2.93-14.07; *P *< .001) [11].

In our particular case, the D1 had a true ostial occlusive lesion and was associated with a 70% lesion in the mid LAD, necessitating double stenting of the bifurcation to salvage the large D1 and protect the diseased LAD.

Based on the previous data, we believe that stenting of bifurcation lesions with two BMSs will significantly increase the risk of restenosis and rate of target vessel revascularization. On the other hand, using two DESs instead will significantly impair re-endothelialization and increase the risk of stent thrombosis. Consequently, we chose to pursue a balanced approach using a hybrid technique of a BMS and a DES. We chose to deploy the BMS at the site of the culprit lesion in the D1 and DES in the LAD. We believe that the BMS at the culprit thrombotic, inflamed site in D1 is more likely to re endothelialize than a DES and the DES in the LAD, is less likely to re-stenose than a BMS. Furthermore, using culotte technique, we were able to completely cover all the coronary layers of the bifurcation lesion, avoiding any excessive "crushing" of displaced and distorted metal against the coronary wall. Thus, in our opinion, we achieved a balance and acceptable compromise between potential benefits and risks. Although this hybrid technique still carries a risk in terms of re-stenosis of the D1 stent and/or stent thrombosis of the LAD stent, we feel this approach could be significantly useful, especially during the acute phase of STEMI where a bifurcation lesion is involved.

In our opinion, the most suitable subsets of bifurcation lesions for this approach should be Medina 0,1,1 and 1,1,1 with significant involvement of the main branch.

## Conclusion

This is the only reported case to our knowledge, where in the setting of an acute ST-segment elevation myocardial infarction, a hybrid culotte technique was successfully performed with excellent long-term results, achieving an acceptable balance of risks between restenosis (in the case of a BMS) and stent thrombosis (in the case of a DES). This case could serve as a sentinel for further research using our rationale in a larger number of patients.

## Consent

Written informed consent was obtained from the patient for publication of this case report and accompanying images. A copy of the written consent is available for review by the Editor-in-Chief of this journal.

## Competing interests

The authors declare that they have no competing interests.

## Authors' contributions

DB, ML, MZ and CD analyzed and interpreted the patient data regarding the unique presentation and the novel intervention performed on the patient. CD, JM and DB were instrumental in obtaining informed consent and also in the preparation of the manuscript. All authors read and approved the final manuscript.
